# An insight into the molecular identity and distribution of *Microhyla taraiensis* (Anura, Microhylidae) in Khyber Pakhtunkhwa, Pakistan

**DOI:** 10.3897/zookeys.1280.172170

**Published:** 2026-05-21

**Authors:** Javed Khan, Muhammad Qayash Khan, Sultan Ayaz, Saman Yaqub, Sarwar Jahan, Abdur Rauf, Jasim Iqbal, Noor Bahadar

**Affiliations:** 1 Department of Zoology, Abdul Wali Khan University, Mardan 23200, Pakistan School of Life Science, Northeast Normal University Changchun China; 2 Department of Zoology, Higher Education, Government of Khyber Pakhtunkhwa, Peshawar, Pakistan Institute of Mountain Hazards and Environment, Chinese Academy of Sciences Chengdu China; 3 Department of Botany, Abdul Wali Khan University, Mardan 23200, Pakistan Department of Zoology, Abdul Wali Khan University Mardan Pakistan; 4 Institute of Mountain Hazards and Environment, Chinese Academy of Sciences, Chengdu, 610041, China Department of Botany, Abdul Wali Khan University Mardan Pakistan; 5 Transgenic Research Center, School of Life Sciences, Northeast Normal University, Changchun, Jilin 130024, China Department of Zoology, Higher Education, Government of Khyber Pakhtunkhwa Peshawar Pakistan

**Keywords:** 16S rRNA, bioacoustics, COI, Khyber Pakhtunkhwa, *Microhyla taraiensis*, mitochondrial DNA, Pakistan, range extension

## Abstract

The taxonomic identity and distribution of Pakistan’s *Microhyla* in Khyber Pakhtunkhwa has not been confirmed at species level using molecular data; historical records relied on morphology alone and often referred to the now-restricted *M. ornata*. A combination of field surveys, morphology, bioacoustics, and mitochondrial DNA was utilized to confirm species identity and map the distribution of *Microhyla taraiensis* in Khyber Pakhtunkhwa (KP), Pakistan. 125 individuals were collected in 13 districts spanning three climatic zones (arid, semi-arid, and hilly/temperate) from lowland plains to lower montane elevations. Adults and tadpoles were examined morphologically; advertisement calls were recorded from one male; and mitochondrial 16S rRNA and COI fragments were sequenced and analyzed with maximum-likelihood phylogenetics. Three COI sequences from Pakistan were identical to one another and matched the *M. taraiensis* mitogenome from Nepal, and the 16S sequence formed a well-supported clade with Nepalese *M. taraiensis*, distinct from *M. ornata* and *M. nilphamariensis*. Verified records document *M. taraiensis* at 19 localities in KP, primarily in shallow, human-modified wetlands (paddy fields, irrigation channels, roadside ditches, floodplain ponds), indicating a wider western distribution along the Himalayan foreland than previously documented. These data constitute the first genetically validated records of *M. taraiensis* in Pakistan, refine regional biogeography at the Indomalayan–Palearctic boundary, and provide a baseline for future genomic, acoustic, and ecological work. Future efforts should pair genome-scale markers with temperature-standardized call libraries, denser spatial sampling, and curated vouchers to assess population structure, refine range limits, and evaluate potential contact with congeners.

## Introduction

The family Microhylidae, commonly known as narrow-mouthed frogs, represents one of the most speciose and geographically widespread radiations of anurans (de Sá et al. 2012). This vast and intricate group comprises more than 700 recognized species allocated to more than 60 genera and 11 or 12 subfamilies, constituting a significant fraction of global amphibian diversity. Their distribution is largely pan-tropical, extending throughout the Americas, sub-Saharan Africa, Madagascar, and Asia from the Indian subcontinent to New Guinea and northern Australia. The family’s major centers of diversity are located in Madagascar, New Guinea, and Southeast Asia, where they have undergone extensive evolutionary radiations (Garg et al. 2019; Belluardo et al. 2022).

Microhylids exhibit remarkable ecological and morphological plasticity, with species adapted to terrestrial, fossorial (burrowing), arboreal, and aquatic lifestyles across a wide spectrum of habitats, from arid deserts to humid tropical rainforests (Rakotoarison et al. 2017; Garg et al. 2019). This ecological breadth is mirrored by a striking diversity in reproductive strategies, which include aquatic larvae with and without feeding mouthparts, as well as direct development, where miniature froglets hatch directly from terrestrial eggs. Despite this functional diversity, many species share a generalized body plan characterized by a globose, teardrop-shaped body, a short snout, and stout hind limbs (Garg et al. 2019). This immense diversity, coupled with a high degree of morphological convergence (homoplasy) driven by parallel adaptations to similar ecological niches, has historically rendered the systematics of Microhylidae exceptionally challenging (Sumida et al. 2000; Scherz et al. 2019). The first and only comprehensive monographic revision of the family was conducted by Parker (1934), a work based largely on osteological characters that remained the foundational taxonomic framework for nearly a century (Gorin et al. 2021). Consequently, significant and reliable progress in untangling the complex phylogenetic relationships within Microhylidae has only been achieved in the modern era through the widespread application of molecular phylogenetic studies. The family’s taxonomic history thus serves as a powerful exemplar of the necessity of genetic data to validate taxonomic and distributional claims, a standard that is now essential for rigorous systematic work within this group.

Within the Asian subfamily Microhylinae, the genus *Microhyla* Tschudi, 1838, is one of the most species-rich taxa, currently comprising more than 50 nominal species of diminutive frogs (Gorin et al. 2021). The genus is widely distributed across Asia, from the Ryukyu Islands of Japan, through China and Southeast Asia, and across the Indian subcontinent to Sri Lanka and Pakistan. The taxonomy of *Microhyla* has been in a state of dynamic flux, characterized by an astonishing rate of species discovery; over half of the currently recognized species have been described within the last 15 years alone (Trofimets et al. 2024). This taxonomic explosion is not merely a consequence of exploring new, remote regions, but is primarily attributable to the widespread adoption of an integrative taxonomic approach. This modern framework, which combines molecular phylogenetics, bioacoustic analysis of advertisement calls, and detailed morphometrics, has consistently revealed that many taxa once considered to be single, widespread species are in fact complexes of multiple, morphologically similar but genetically distinct “cryptic” species (Garg et al. 2019; Belluardo et al. 2022). These studies have collectively established a predictable pattern within the genus: the broader the purported geographic range of a single, morphologically defined species, the higher the likelihood that it represents a species complex. This creates a strong *a priori* scientific basis for subjecting any population found at the periphery of a known range, or in a geographically disjunct location, to rigorous genetic scrutiny to validate its taxonomic identity.

The amphibian fauna of Pakistan remains relatively understudied in comparison to neighboring biodiversity-rich regions. Current checklists record approximately 24 anuran species belonging to four families: Bufonidae, Dicroglossidae, Megophryidae, and Microhylidae (Safi and Karl 2024). For decades, historical faunal lists for Pakistan have included a single representative of the genus *Microhyla*, *Microhyla ornata*. However, this long-standing record now stands in direct contradiction to robust, recent molecular evidence. The comprehensive systematic revision of South Asian *Microhyla* by Garg et al. (2019) conclusively demonstrated that the distribution of true *M. ornata* is restricted to Peninsular India and Sri Lanka (Garg et al. 2019). This finding renders all previous records of “*M. ornata*” from northern India, Nepal, Bangladesh, and, by extension, Pakistan, taxonomically obsolete.

The true specific identity of the *Microhyla* population(s) in Khyber Pakhtunkhwa had not been confirmed at species level with molecular vouchers, and the long-standing *M. ornata* record for the province therefore remained in need of species-level resolution. The recent collection of specimens morphologically resembling members of the *M. ornata* group in Khyber Pakhtunkhwa provides the material basis to confirm species identity with molecular evidence. The present study provides the first genetically and morphologically validated identification of *M. taraiensis* in KP, correcting the long-standing *M. ornata* record for the province and documenting a significant westward range extension for the species. To provide an unambiguous species-level identification, DNA barcoding has become an indispensable tool in modern amphibian systematics (Vences et al. 2005; Chambers and Hebert 2016). Given the profound taxonomic uncertainty surrounding the genus *Microhyla* in Pakistan, and in light of the recent discovery of a population of these frogs in Khyber Pakhtunkhwa, this study was undertaken to provide the definitive identification of this lineage. The principal aim of this research is to provide the genetic confirmation of *Microhyla taraiensis* in Khyber Pakhtunkhwa, Pakistan, and to document a westward range extension for the species beyond its previously confirmed Nepalese range.

## Materials and methods

### Study area

The study was conducted across Khyber Pakhtunkhwa (KP) province in northern Pakistan, immediately west of the Islamabad Capital Territory. Fieldwork took place from September 2021 to November 2023 at 19 sites encompassing shallow waterbodies and agricultural landscapes. Sampling sites were distributed across 13 districts spanning a broad elevational and climatic gradient, grouped here as hilly/temperate (Abbottabad, Mansehra, Torghar, Battagram, Buner, Upper Dir, Lower Dir), semi-arid (Mardan, Swabi, Charsadda), and arid (Hangu, Karak, Lakki Marwat). Higher elevations are characterized by cool–temperate conditions, whereas lower valleys and plains are predominantly subtropical.

### Sampling

We surveyed each locality using standardized nocturnal time-constrained Visual Encounter Surveys (VES) combined with auditory call surveys (Shaffer et al. 1994). Searches focused on shallow waterbodies and adjacent microhabitats (e.g., paddy fields, irrigation channels, roadside ditches, temporary pools, and pond margins). At each site we made at least one post-dusk survey during the peak calling period; observers moved slowly along the water’s edge using headlamps and hand/dip nets, with brief listening stops to detect calling males. For every encounter we recorded GPS coordinates, date/time, habitat, and microhabitat.

Individuals were captured by hand or with dip nets, photographed in life, and examined for external characters. Morphological identifications followed regional keys and comparative diagnoses, with reference to the original description and subsequent treatments of *Microhyla taraiensis*. Across all visits, we collected 125 *Microhyla* individuals at 19 sites in 13 districts. Most animals were measured and released at the point of capture after documentation.

To obtain voucher-backed genetic material, a representative subset of adults and tadpoles from each climatic zone was retained. Specimens were humanely euthanized using an overdose of buffered tricaine methanesulfonate (MS-222; ≥ 1 g L^-1^ for adults, ≥ 0.5 g L^-1^ for tadpoles), following contemporary animal-care guidelines for amphibians. From each euthanized specimen, a small sample of thigh muscle (adults) or tail/axial muscle (tadpoles) was dissected and preserved in ≥ 95% ethanol in sterile microcentrifuge tubes; ethanol was refreshed within 24 h, and tissues were later stored at –20 °C pending DNA extraction. Whole-body vouchers were fixed in 10% neutral-buffered formalin for 24–48 h and transferred to 70% ethanol. Voucher specimens and associated tissue samples are deposited in the herpetological collection of the Department of Zoology, Abdul Wali Khan University Mardan; catalog numbers will be provided upon formal accession.

All work complied with national regulations; collections were conducted under permits issued by the Khyber Pakhtunkhwa Wildlife Department, in accordance with the institutional animal-use guidelines of Abdul Wali Khan University Mardan. Field equipment was rinsed and wiped with 70% ethanol between specimens to minimize cross-contamination, and handling time was kept as brief as possible to reduce stress.

### Morphological measurements

Adult morphology was quantified using digital calipers (0–150 mm range, 0.01 mm resolution; accuracy ± 0.02 mm). Calipers were checked against gauge blocks before each session. All measurements were taken to the nearest 0.01 mm on the left side of the body with joints gently extended, and each metric was recorded three times and averaged to reduce measurement error. Small features (e.g., disc widths, tubercles) were measured under a dissecting microscope using calipers/ocular scale as appropriate. All measurements follow standard anuran protocols used for *Microhyla* by other researchers (Trofimets et al. 2024).

### Advertisement call recording and analysis

Calling males were located at night by auditory surveys; the focal recording presented here was made at 23:15 local time. Advertisement calls were recorded using an A70 itel smartphone (built-in microphone) in video mode. After locating a calling male, we obtained three call bouts at ~10 cm from the snout with the microphone oriented toward the caller; the best uninterrupted segment selected for analysis was 16.14 s. We avoided handling or illuminating the frog during recording and selected segments free of audible wind or handling noise. The audio track was extracted from the original smartphone video, downmixed from stereo to mono, and converted to uncompressed 16-bit PCM WAV at 44,100 Hz using macOS afconvert (no filtering, normalization, or noise reduction applied); this file is deposited as Suppl. material 1. Oscillograms and spectrograms were generated in AudioMass (v. 1.0, web application) and inspected with an online spectrum analyzer. Spectrograms were computed with a Hann window, 1024-point FFT, and 50% overlap. The built-in microphone of the recording device has a limited frequency response (typically 100 Hz to 8 kHz), which may not capture the full spectral range of the call; all acoustic parameters should therefore be treated as approximate and compared with caution against recordings made with calibrated equipment.

### Molecular analyses

Genomic DNA was extracted from ethanol-preserved skeletal muscle (adults) or axial/tail muscle (tadpoles) using a salt (ammonium acetate) precipitation protocol (Bruford et al. 1998). Extraction blanks were included to monitor contamination. DNA quality and concentration were checked by agarose gel electrophoresis (1.0–1.5%) and spectrophotometry. We targeted two mitochondrial markers widely used in anuran systematics: 16S rRNA and COI. The 16S fragment was amplified with primers 16Sar-L (5'-CGCCTGTTTATCAAAAACAT-3') and 16Sbr-H (5'-CCGGTCTGAACTCAGATCACGT-3'), and COI with LCO1490 (5'-GGTCAACAAATCATAAAGATATTGG-3') and HCO2198 (5'-TAAACTTCAGGGTGACCAAAAAATCA-3'), following established protocols (Trofimets et al. 2024). PCRs (25 µL) contained ~20–50 ng template DNA, 1X buffer, 1.5–2.5 mM MgCl_2_, 200 µM each dNTP, 0.3 µM each primer, and 0.5–1.0 U Taq DNA polymerase. Cycling profiles were initial denaturation 95 °C for 2–3 min; 35 cycles of 95 °C for 30 s, annealing at 50–52 °C (16S) or 46–48 °C (COI) for 30 s, 72 °C for 60 s; final extension 72 °C for 5–10 min. Amplicon size was verified on agarose gels against ladder.

PCR products were purified with a silica column-based PCR purification kit (Thermo Fisher Scientific) and sequenced bidirectionally by Macrogen (Seoul, South Korea) using the amplification primers. Chromatograms were inspected in FinchTV v. 1.4; low-quality ends and ambiguous bases were trimmed, and forward/reverse reads were assembled into consensus sequences in BioEdit v. 7.2.5. The sequences reported here are the subset that yielded clean bidirectional chromatograms for the target loci; amplification success was not tabulated separately. Preliminary identifications were obtained by BLASTn searches against NCBI nucleotide databases to retrieve top matches and reference sequences from named congeners. Alignments for 16S and COI were made separately in MEGA11 (v. 11.0.13) using MUSCLE with default gap penalties; poorly aligned terminal regions were trimmed to a common length. The best-fit nucleotide substitution model for each gene was selected by BIC in MEGA. Phylogenetic relationships were inferred with Maximum Likelihood and 1,000 nonparametric bootstrap replicates. The trees shown in the main text are midpoint-rooted; exploratory analyses rooted with a related microhylid reference taxon (*Kaloula pulchra*) were used only as topology checks and recovered the same major relationships. Uncorrected pairwise p-distances (within/between species) were computed in MEGA to contextualize divergences relative to common amphibian barcoding thresholds.

## Results

### Morphometric analysis of tadpole and adults

**Figure 1.**
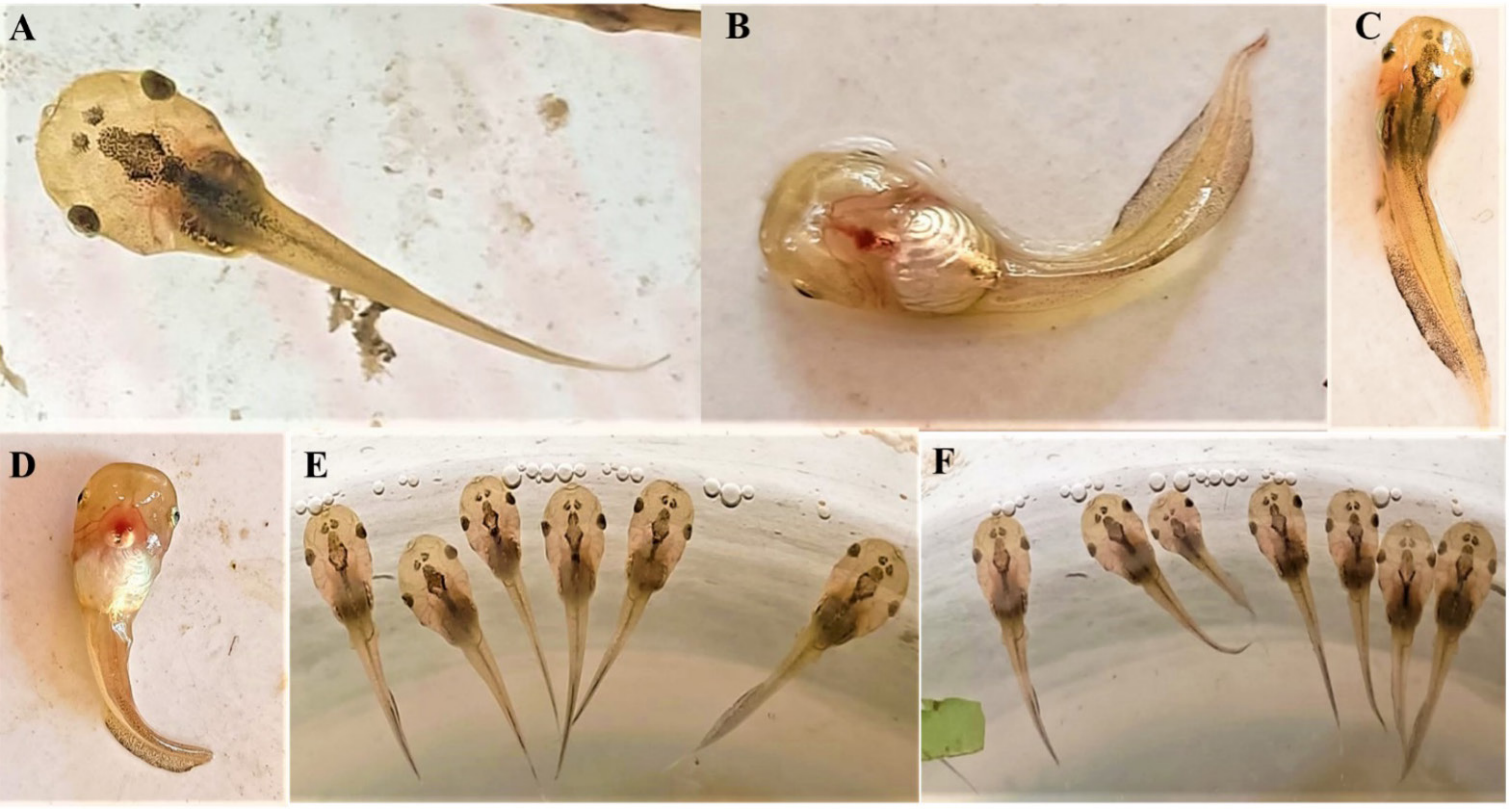
The tadpoles of *Microhyla taraiensis*. **A, C**. Dorsal view; **B, D**. Ventral view with black-dotted tail fin; **E, F**. Tadpoles in pond.

Tadpoles attributed to *Microhyla taraiensis* were translucent olive-cream with a silvery belly (Fig. 1). Through the dorsal body wall the brain and axial musculature/notochord were visible; the ventral side showed a conspicuous, blood-filled heart and major vessels. The head was rounded; snout rounded; nares small and close to the eyes; eyes lateral with a golden iris and black pupil. The tail was transparent with distinct dorsal and ventral fins bearing scattered black punctations. The vent tube was median. The mouth was small and terminal with keratinized jaw sheaths; labial tooth rows were reduced or not externally discernible. Gosner stages were not recorded for the material described here, so comparisons are restricted to general external morphology.

**Figure 2.**
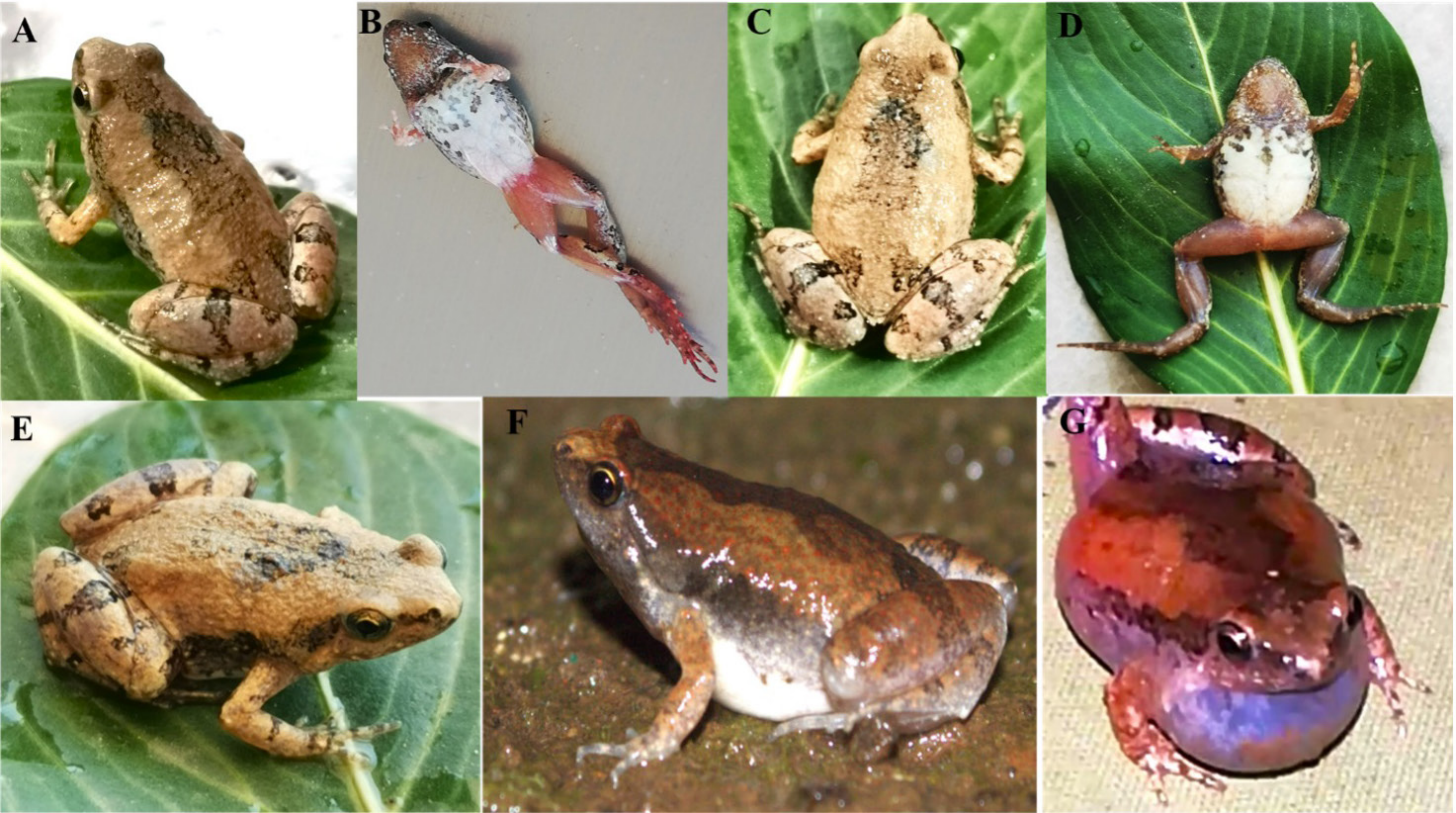
The adult of *Microhyla taraiensis*. **A**. Dorsal view of male frog; **B**. Ventral view of male frog; **C**. Dorsal view of female frog; **D**. Ventral view of female frog; **E**. Prominent golden iris, black pupil and round nostril of frog; **F**. Visible red dots on the skin; **G**. Male frog with bluish vocal sac.

The adult’s live coloration and pattern: dorsum light brown with small reddish dots (diminished on the metatarsus and foot), a rectangular dark mark across the interorbital region, and two longitudinal dark stripes extending from the orbital region toward the groin. Ventrum cream with fine black spotting. Females had a pale to brownish throat; males showed a conspicuously darker, brown to blackish gular region consistent with a median subgular vocal sac. Of the individuals retained for detailed morphometric description, six adults were examined (♀ *n* = 2, ♂ *n* = 4); females were larger than males in this sample. Head relatively broad; snout short and truncate; canthus rostralis indistinct; eyes small and lateral with golden iris and black pupil; tympanum not discernible externally, concealed beneath the supratympanic fold; fold present but indistinct, consistent with the original description of*M. taraiensis* (Howlader et al. 2015); tongue elliptical. Maxillary and vomerine teeth absent. Forelimbs and hands: finger length formula F1 < F2 < F4 < F3; nuptial pads were not observed on the examined individuals. Inner metacarpal tubercle large, rounded, and flattened; outer metacarpal tubercle smaller. Hind limbs and feet: hind limbs robust; toe tips rounded; toe length formula T1 < T2 < T5 < T3 < T4 (Fig. 2).

### Molecular identification and characterization

**Figure 3.**
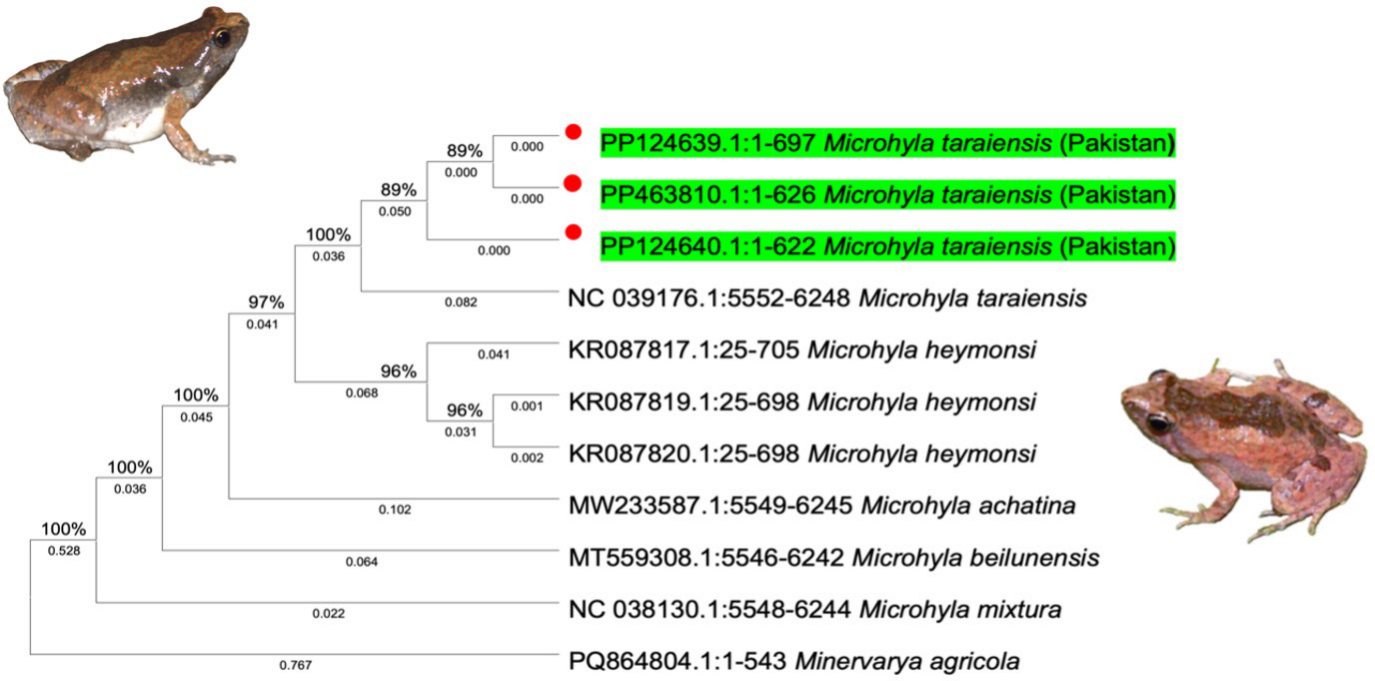
Maximum-likelihood COI phylogeny showing Pakistani *Microhyla taraiensis* clustering with reference *M. taraiensis* and separated from the other sampled taxa. The tree is midpoint-rooted; numbers at nodes indicate bootstrap support.

The three Pakistani COI sequences (PP124639, PP124640, PP463810) were identical to one another (no variable sites across the amplified fragment). BLASTn searches returned *Microhyla taraiensis* as the top match, with 100% identity to the corresponding COI region of the complete mitochondrial genome from Nepal (NC_039176). High-scoring hits also included sequences labeled *M. heymonsi* from Laos (KR087819, KR087820), reflecting the limited discriminatory power of short COI fragments in some *Microhyla*. In the maximum-likelihood tree (Fig. 3), our COI sequences clustered within the *M. taraiensis* clade and did not group with *M. heymonsi*.

**Figure 4.**
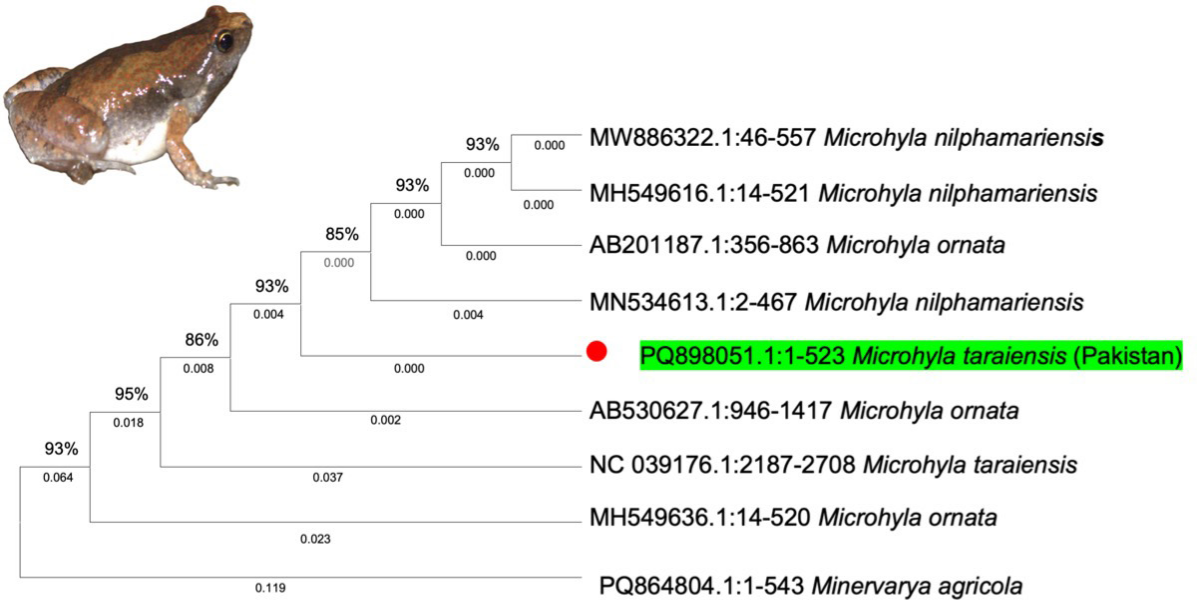
Maximum-likelihood 16S rRNA phylogeny showing the Pakistani *Microhyla taraiensis* sequence clustering with Nepalese *M. taraiensis* and separated from *M. ornata* and *M. nilphamariensis*. The tree is midpoint-rooted; numbers at nodes indicate bootstrap support.

The 16S rRNA sequence generated from Pakistan (PQ898051) showed highest BLASTn similarity to *M. taraiensis* from Nepal (NC_039176; KY655952–KY655953) and lower similarity to members of the *M. ornata* complex, including *M. ornata* (MH549636) and *M. nilphamariensis* (MH549616). In the 16S phylogeny (Fig. 4), the Pakistani sequence formed a well-supported clade with Nepalese *M. taraiensis*, clearly separated from *M. ornata* and *M. nilphamariensis*. Taken together, COI and 16S evidence are congruent and support assignment of the Pakistani material to *Microhyla taraiensis*.

### Vocalization

**Figure 5.**
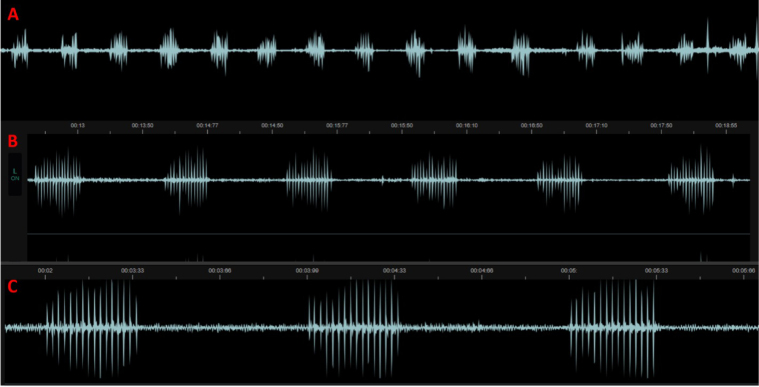
Advertisement call structure of *M. taraiensis* A) 14 notes from the 16-second recorded segment, B) intervals between notes, C) pulses in each note.

**Figure 6.**
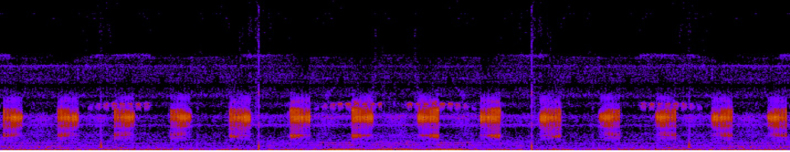
A Spectrogram of recorded *M. taraiensis* sound showing 14 notes.

Advertisement calls were recorded at Khar Colony, Adina (Swabi District), Khyber Pakhtunkhwa, Pakistan (34.2026933°N, 72.2707875°E) on 15 July 2023 at 23:15 local time. Males began calling shortly after dusk and were clearly audible under quiet conditions; the focal male was recorded at ~10 cm microphone distance. Calls consisted of a sequence of short, pulsed notes rather than a continuous trill. Representative oscillogram and spectrogram are shown in Figs 5, 6.

From a 16-s recording segment of one male, we analyzed 14 notes (note rate ≈ 0.88 notes s^-1^). Each note contained 15 or 16 pulses. Mean inter-note interval (peak-to-peak, excluding note duration) was 0.704 ± 0.156 s (range 0.61–1.25 s; *n* = 14), and mean note duration was 0.337 ± 0.014 s (range 0.31–0.38 s). Given 15–16 pulses per ~0.34-s note, the pulse rate is approximately 46 pulses s^-1^. The resulting duty cycle averaged ~0.32. Because recordings were made with a smartphone (automatic gain control) and ambient temperature at the caller was not recorded, we do not report absolute amplitude or temperature-standardized frequency values; spectrograms are provided for qualitative comparison only. Onomatopoeic description provided in the field notes (“kar, kar, kar”) matches the short, pulsed notes observed in the spectrograms. The analyzed 16.14-s recording is provided as Suppl. material 1.

### Distribution

**Figure 7.**
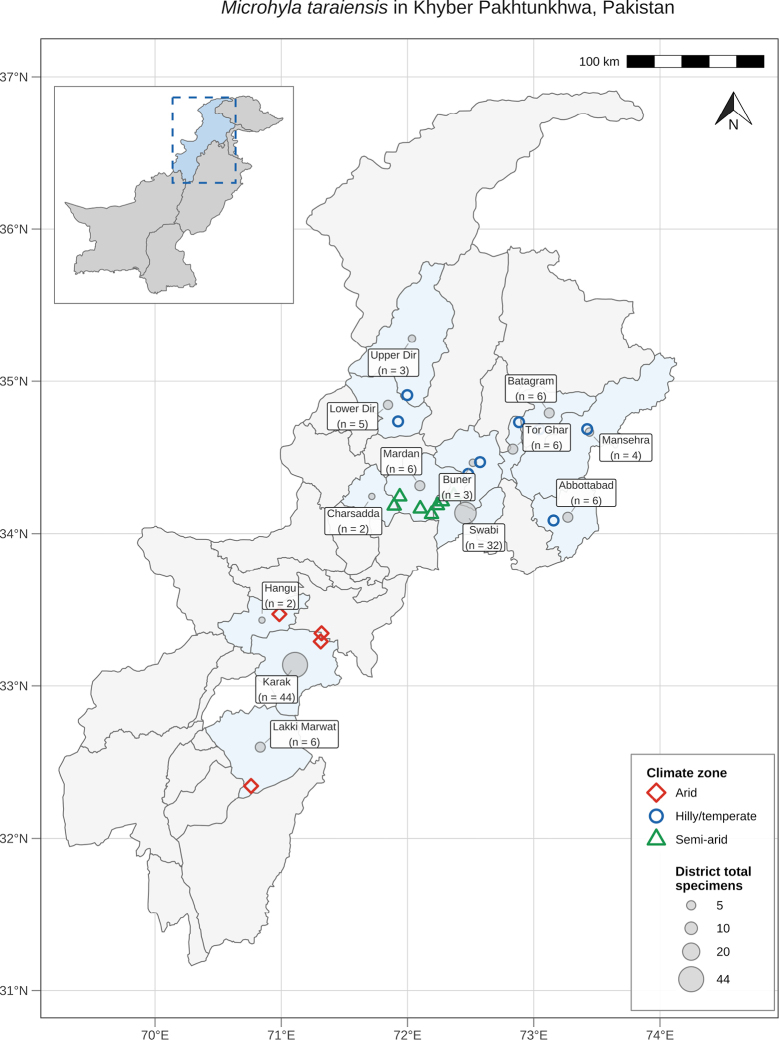
Distribution and climatic zonation of *Microhyla taraiensis* across Khyber Pakhtunkhwa, Pakistan. Verified collection localities (*n* = 19 sites across 13 districts) are plotted as hollow symbols color-coded by climate zone: diamonds (arid, red), circles (hilly/temperate, blue), and triangles (semi-arid, green). Grey bubbles indicate district-level specimen totals (proportional to area). The inset map shows the position of Khyber Pakhtunkhwa within Pakistan. Coordinate grid lines are drawn at 1° intervals (WGS 84).

We documented *Microhyla taraiensis* at 19 sites across 13 districts of Khyber Pakhtunkhwa (Abbottabad, Mansehra, Torghar, Battagram, Buner, Upper Dir, Lower Dir, Mardan, Swabi, Charsadda, Hangu, Karak, and Lakki Marwat), spanning lowland agro-mosaic landscapes through foothill and lower montane districts (Fig. 7). Detections came from shallow waterbodies and adjacent habitats typical for the genus (paddy fields, irrigation channels, roadside ditches, floodplain ponds), and included all three climatic zones defined for the study (arid, semi-arid, and hilly/temperate). Table 1 provides an overview of the collections. Molecular data (COI and 16S rRNA) from representative localities support assignment of the sequenced specimens to *M. taraiensis* sensu the Nepalese reference sequences; conspecificity across all surveyed sites is inferred from morphological consistency with the sequenced material. These results support a single taxon across the surveyed portion of KP. From a regional perspective, KP lies along the contact zone between the Indomalayan (Oriental) and Palearctic zoogeographic regions. Our verified records occur within the Indomalayan sector of the province or near this boundary. To our knowledge, these are the first genetically validated records of *M. taraiensis* from Pakistan and the first province-wide assessment for this species in KP, indicating a broader western distribution along the Himalayan foreland than previously documented. We caution that historical “*Microhyla ornata*” records in Pakistan may include *M. taraiensis*; voucher-backed genetic data will be needed to refine species limits and potential contact with related congeners outside the current sampling area.

**Table 1.** Sampling districts, sampling area, latitude, longitude, temperature, humidity, and number of specimens of *M. taraiensis*.

Sampling Districts	Sampling area	Coordinates	Temperature (°C)	Humidity (%)	Specimens (collected)
Abbottabad	Malkan road	34.0869221°N, 73.1569265°E	27	74%	6
Karak	Krapa	33.3445999°N, 71.3191576°E	34	46%	25
Karak	Karak (Has)	33.290865°N, 71.3128476°E	32	50%	19
Dir Upper	Shaif Alam	34.9084962°N, 71.9981102°E	34	61%	3
Dir Lower	Sarai Bala	34.7362767°N, 71.925358°E	30	51%	5
Hangu	Togh Bala	33.4698698°N, 70.9849572°E	34	35%	2
Lakki Marwat	Pezu	32.343832°N, 70.760833°E	32	47%	6
Mansehra	Hangari	34.685483°N, 73.423383°E	26	70%	4
Battagram	Battagram Has	34.684729°N, 73.045177°E	28	59%	6
Torghar	Faisal Mosque	34.731121°N, 72.883475°E	33	52%	6
Charsadda	Dargi	34.18341°N, 71.89452°E	33	41%	2
Mardan	Jamra	34.2434529°N, 71.9386182°E	25	38%	2
Mardan	Ghumbat	34.12829°N, 72.190274°E	30	32%	3
Mardan	Khana Kali	34.164427°N, 72.1011°E	27	33%	1
Swabi	Adina	34.211611°N, 72.275424°E	28	39%	18
Swabi	Nazar Kalay	34.185917°N, 72.236972°E	29	40%	10
Swabi	Shewa	34.2499°N, 72.36692°E	31	49%	4
Buner	Ambela	34.392456°N, 72.48062°E	29	68%	2
Buner	Dewana Baba	34.46948°N, 72.574691°E	28	51%	1

## Discussion

Our data provide the first genetically validated evidence that *Microhyla taraiensis* occurs widely in Khyber Pakhtunkhwa (KP), Pakistan, spanning 19 sites across 13 districts and multiple climatic zones. This result extends the confirmed western range limit of the species from the Terai plains of Nepal into northwestern Pakistan, consistent with the continuity of lowland and foothill habitats along the Himalayan foreland (Howlader et al. 2015; Khatiwada et al. 2017). Given KP’s position near the boundary between the Indomalayan (Oriental) and Palearctic zoogeographic regions, our findings suggest that *M. taraiensis* occupies the Indomalayan sector of the province and may reach the contact zone in valley bottoms. Historically, many Pakistani microhylid records were assigned to “*ornata*” without genetic vouchers, and our results indicate that some of those records may represent *M. taraiensis*. Resolving that legacy will require targeted re-sampling and the re-examination of archived material (Akram et al. 2021). Within the broader South Asian biogeographic context, *M. taraiensis* differs from its closest relative in the ornata complex, *M. nilphamariensis*, described from the lowland Terai of northern Bangladesh and reported from adjacent northeastern India (Howlader et al. 2015), though its full range remains incompletely documented; the westward extension of *M. taraiensis* confirmed here suggests that *M. taraiensis* is the member of the complex best suited to the drier, more seasonal conditions of the Himalayan foreland. Globally, *M. taraiensis* is currently listed as Least Concern on the IUCN Red List; however, this assessment predates the verified western range data reported here. Given that KP populations depend on small, human-modified wetlands susceptible to agricultural intensification, pesticide application, and channel concreting, local vulnerability merits monitoring and the range extension documented here should inform any future reassessment.

Morphology of adults and larvae in KP is congruent with diagnoses for *M. taraiensis* and closely related congeners in the *M. ornata* complex. Adults are small, with females slightly larger than males (♀ 19.0–23.2 mm SVL; ♂ 18.3–20.12 mm SVL), and show the expected sexual trait of a darker gular region in calling males. Dorsal patterning (small reddish dorsal punctations, interorbital mark, and dark longitudinal streaks), hand and foot tubercles, and finger–toe length formulas (F1 < F2 < F4 < F3; T1 < T2 < T5 < T3 < T4) match published variation (Howlader et al. 2015; Garg et al. 2019). As in other *Microhyla*, tadpoles were translucent with visible brain and notochord and bore keratinized jaw sheaths; external labial tooth rows are reduced and can be difficult to discern, which can lead to field notes describing a “toothless” oral disc (diagnostic at family level rather than species-specific). These consistencies strengthen the case that our genetically assigned material is morphologically typical for *M. taraiensis*.

Acoustic evidence, although limited to a single focal male, is also consistent with expectations for small microhylids: short, pulsed notes delivered at low duty cycle. We report note structure (≈15 or 16 pulses per ~0.34 s note; inter-note intervals ~0.61–1.25 s), but we avoid absolute amplitude and temperature-standardized frequency because recordings were made with a smartphone microphone under automatic gain control and ambient temperature was not recorded; following the standards recommended by others (Köhler et al. 2017). A larger, temperature-controlled dataset with calibrated recording equipment and standardized FFT parameters would enable direct comparison to calls from topotypic *M. taraiensis* and sympatric congeners, which is an important next step for integrative taxonomy.

Genetically, both markers analyzed support assignment to *M. taraiensis*. COI sequences from Pakistan were identical to each other and returned *M. taraiensis* as top BLAST matches against the Nepal mitogenome (NC_039176). We note that short COI fragments can show limited resolving power within *Microhyla*, occasionally returning high identity to *M. heymonsi* in database searches an artifact of marker choice rather than true affinity. By contrast, 16S rRNA placed our material with Nepalese *M. taraiensis* and apart from the *M. ornata* complex (*M. ornata*, *M. nilphamariensis*), which aligns with the widespread use of 16S in amphibian systematics (Vences et al. 2005; Vieites et al. 2009; Akram et al. 2021). Together, these lines of evidence support a single taxon across our KP sampling extent.

Ecologically, detections came from shallow, human-modified wetlands (paddy fields, irrigation channels, roadside ditches, and floodplain ponds), which are normal breeding sites for *Microhyla*. The prevalence of records in agro-mosaic landscapes suggests that *M. taraiensis* can tolerate moderate disturbance and exploit ephemeral waters produced by monsoon rainfall and irrigation. Nonetheless, its reliance on small, fish-free waterbodies implies sensitivity to pesticide regimes, water abstraction, and the concreting of drainage channels. A province-wide view, as provided here, is a useful baseline for tracking future changes in occupancy as agricultural practices and climate fluctuate.

Our study provides informed priorities for future work. Morphometric inference is constrained by the small adult sample (*n* = 6; ♀ *n* = 2), and we therefore emphasize descriptive statistics and %SVL profiles rather than formal sex-difference tests or unstable multivariate ordinations. The acoustic dataset is from a single male without temperature annotation, limiting cross-study comparability. Genetically, we targeted standard mtDNA markers (16S, COI); adding nuclear loci or genome-wide markers (e.g., ddRAD/target capture) would permit tests of population structure, gene flow, and potential contact with related lineages at the eastern and southern margins of KP (Harrison 1989; Glon et al. 2021; Vaux et al. 2023). Finally, although our records span 13 districts, sampling was biased toward accessible waterbodies; high-elevation valleys, protected areas, and arid districts at the western fringe merit additional surveys.

Despite these caveats, the convergence of morphology, bioacoustics, and mtDNA strongly supports the presence and broad distribution of *M. taraiensis* in KP. This finding refines the western biogeography of the *M. ornata* complex sensu lato and underscores the importance of voucher-linked genetics for small, cryptic anurans that are prone to historical misidentification. We recommend (i) continued, standardized nocturnal surveys across seasons; (ii) deposition of tissue vouchers and whole-body specimens with catalog numbers in institutional collections; (iii) expansion of call libraries with temperature and calibration metadata; and (iv) integrative analyses incorporating genomic data; and (v) public archiving of georeferenced records, audio files, images, sequence alignments, and analysis scripts. Such efforts will sharpen regional species limits, clarify range boundaries relative to the Indomalayan–Palearctic transition, and provide an evidence base for conservation assessments and environmental decision-making in northern Pakistan.

## Conclusions

Our study provides the first genetically validated records of *Microhyla taraiensis* in Khyber Pakhtunkhwa, Pakistan and shows that the species is widely distributed across Khyber Pakhtunkhwa (19 sites, 13 districts) in agro-mosaic lowlands and foothill habitats. Morphological traits of adults and larvae, together with call structure from a focal male, are congruent with *M. taraiensis* diagnoses. Mitochondrial markers (16S rRNA and COI) place the Pakistani material with Nepalese *M. taraiensis* and apart from members of the *M. ornata* complex, clarifying long-standing uncertainty created by morphology-only records. The confirmed presence of *M. taraiensis* along the Indomalayan margin of KP refines the western range of the species and establishes a baseline for biogeographic inference and conservation planning at the Indomalayan–Palearctic interface.

## Additional information

### Artificial Intelligence (AI) use

Regarding the use of AI in the preparation of this manuscript, the authors declare the following:

ChatGPT-5 was used for English grammar correction. The authors are taking full responsibility.

### Author ORCIDs

J. Khan https://orcid.org/0009-0008-8765-9487

M. Q. Khan https://orcid.org/0000-0002-3067-1740

S. Ayaz https://orcid.org/0009-0006-7400-4890

S. Yaqub https://orcid.org/0009-0006-1933-6315

S. Jahan https://orcid.org/0000-0001-8056-7485

A. Rauf https://orcid.org/0000-0002-5113-8400

J. Iqbal https://orcid.org/0000-0001-8242-1383

N. Bahadar https://orcid.org/0000-0001-9576-5345

### Data availability

All of the data that support the findings of this study are available in the main text or Supplementary Information.

## Supplementary materials

Supplementary material 1 Supplementary audio Data type 7z Explanation note Advertisement call of *Microhyla taraiensis*, recorded at Khar Colony, Adina, Swabi District, Khyber Pakhtunkhwa, Pakistan (34.2026933°N, 72.2707875°E; WGS 84) on 15 July 2023 at 23:15 h (PKT, UTC+5) by Javed Khan. Single focal male recorded at ~ 10 cm from the built-in microphone of an A70 itel smartphone. Duration 16.14 s; 44,100 Hz; 16-bit PCM WAV; mono; no post-processing applied. Fourteen notes were analyzed from this segment; full acoustic parameters are given in the main text and in the accompanying metadata file. Ambient temperature at the time of recording was not measured. Files: Microhyla_taraiensis_KP_Pakistan_Swabi_15Jul2023_advertisement_call.wav; Microhyla_taraiensis_KP_Pakistan_Swabi_15Jul2023_advertisement_call_METADATA.txt https://binary.pensoft.net/file/1652673This dataset is made available under the Open Database License (http://opendatacommons.org/licenses/odbl/1.0/). The Open Database License (ODbL) is a license agreement intended to allow users to freely share, modify, and use this Dataset while maintaining this same freedom for others, provided that the original source and author(s) are credited. Javed Khan, Muhammad Qayash Khan, Sultan Ayaz, Saman Yaqub, Sarwar Jahan, Abdul Rauf, Jasim Iqbal4, Noor Bahadar
